# A conserved leucine occupies the empty substrate site of LeuT in the Na^+^-free return state

**DOI:** 10.1038/ncomms11673

**Published:** 2016-05-25

**Authors:** Lina Malinauskaite, Saida Said, Caglanur Sahin, Julie Grouleff, Azadeh Shahsavar, Henriette Bjerregaard, Pernille Noer, Kasper Severinsen, Thomas Boesen, Birgit Schiøtt, Steffen Sinning, Poul Nissen

**Affiliations:** 1 Danish Research Institute of Translational Neuroscience—DANDRITE, Nordic-EMBL Partnership for Molecular Medicine, Department of Molecular Biology and Genetics, Aarhus University, Gustav Wieds Vej 10C, Aarhus C DK-8000, Denmark; 2 Translational Neuropsychiatry Unit, Department of Clinical Medicine, Aarhus University, Skovagervej 2, Risskov DK-8240, Denmark; 3 inSPIN and iNANO centers, Department of Chemistry, Aarhus University, Langelandsgade 140, Aarhus C DK-8000, Denmark

## Abstract

Bacterial members of the neurotransmitter:sodium symporter (NSS) family perform Na^+^-dependent amino-acid uptake and extrude H^+^ in return. Previous NSS structures represent intermediates of Na^+^/substrate binding or intracellular release, but not the inward-to-outward return transition. Here we report crystal structures of *Aquifex aeolicus* LeuT in an outward-oriented, Na^+^- and substrate-free state likely to be H^+^-occluded. We find a remarkable rotation of the conserved Leu25 into the empty substrate-binding pocket and rearrangements of the empty Na^+^ sites. Mutational studies of the equivalent Leu99 in the human serotonin transporter show a critical role of this residue on the transport rate. Molecular dynamics simulations show that extracellular Na^+^ is blocked unless Leu25 is rotated out of the substrate-binding pocket. We propose that Leu25 facilitates the inward-to-outward transition by compensating a Na^+^- and substrate-free state and acts as the gatekeeper for Na^+^ binding that prevents leak in inward-outward return transitions.

## Abstract (web summary)

Neurotransmitter:sodium symporters (NSS) perform Na^+^-dependent amino-acid uptake, but a full structural description of their function is lacking. Here the authors present a structure of the bacterial NSS LeuT in the outward-oriented empty state and show that a Leu residue occupies the empty substrate site and is essential for function.

## Graphical abstract

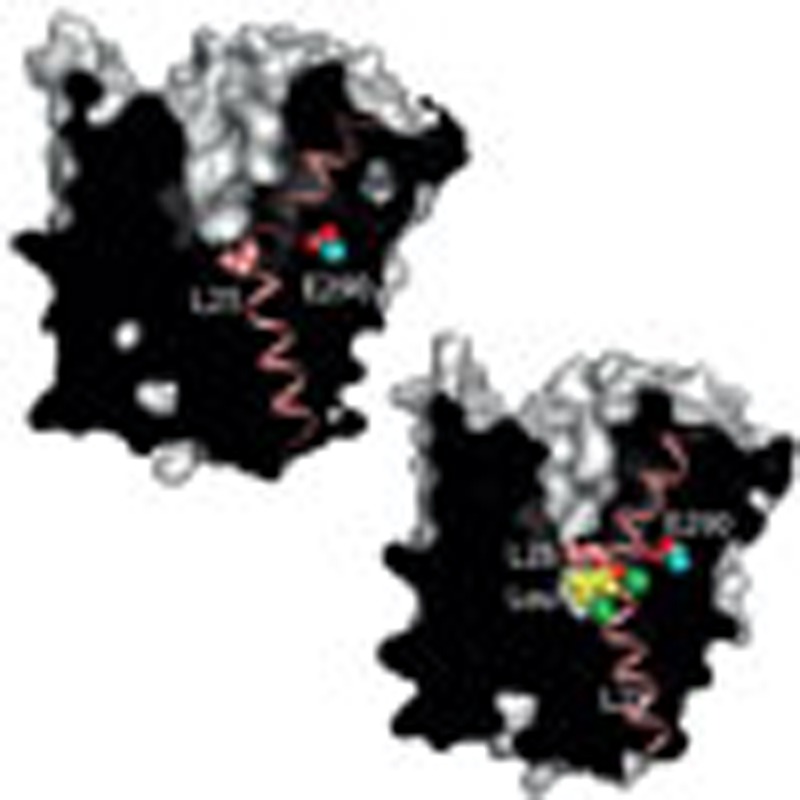

Neurotransmitter:sodium symporters (NSS) terminate the signal transmission in brain by active reuptake of neurotransmitters from the synaptic cleft through a Na^+^-dependent mechanism. NSS include targets for pharmaceuticals like antidepressants, as well as psychostimulants. The family also encompasses a large group of eukaryotic and prokaryotic amino-acid transporters^1^. The main body of structural information for the NSS family comes from the *Aquifex aeolicus* amino-acid transporter LeuT with crystal structures of Na^+^-bound, outward-oriented states^2^, also with bound substrates at a central S1 site^3^ and inhibitors bound at a hydrophobic, extracellular cavity^4^,^5^. The structure of a eukaryotic NSS, the *Drosophila melanogaster* dopamine transporter (dDAT) also represents an outward-oriented, Na^+^-bound state with bound inhibitors or substrate^6^,^7^ and shows close resemblance to LeuT indicating common mechanisms of transport. Similarly, the introduction of mutations copying the human serotonin transporter (hSERT) renders LeuT sensitive to SERT-specific inhibitors^8^. In NSS, two Na^+^-binding sites (Na1 and Na2) as well as a substrate-binding site (S1) are situated approximately in the middle of the membrane, centred on unwound regions of transmembrane helix 1 (TM1) and TM6 (ref. 3). The TM1 unwound region is involved in the direct coordination of both Na1 and Na2 ions and facilitates a crosstalk between the Na2 site and S1, where bound substrate also interacts directly with Na^+^ at the Na1 site^3^. LeuT structure has also been determined in an inward-open apo state^2^, whereas recently reported structures of the hydrophobic amino-acid transporter (MhsT) from *Bacillus halodurans* revealed an occluded, inward-oriented state with bound Na^+^ and substrate^9^. The MhsT structure also presented a possible mechanism for the outward-to-inward transition and Na^+^ solvation from the cytoplasmic side through unwinding and flexibility of the intracellular part of TM5, which is structurally and functionally linked to the extracellular closure of the hydrophobic cavity^9^.

All combined, current structural information depicts a consistent mechanism of Na^+^-gradient-dependent binding and occlusion of extracellular Na^+^ and substrate followed by a switch to the inward-oriented state and intracellular opening and release of Na^+^ and substrate. However, no structural information is available on the Na^+^- and substrate-free return steps associated with the inward-to-outward transition of the NSS family. This transition is especially intriguing because it takes place without Na^+^ and substrate, so how will it proceed from the inward-open state and is it sensitive to the Na^+^ gradient?

The eukaryotic NSS are also Cl^−^-dependent^6^,^7^,^10^,^11^,^12^, which is released with Na^+^ and substrate on the intracellular side, whereas the bacterial transporters use a negatively charged residue, for example, Glu290 in LeuT^13^,^14^,^15^,^16^, which most likely is also the site of H^+^ counter-transport in the inward-to-outward transition^14^,^15^,^16^. Some eukaryotic NSSs, such as the serotonin transporter (SERT) counter-transport K^+^ but can also accept H^+^ (ref. 17). Notably, the inward-to-outward return is rate limiting for the overall transport rate of SERT^18^ supporting the notion that this transition is particularly challenging.

To address this fundamental aspect of NSS function we purified LeuT in a Na^+^- and substrate-free state. Aiming for an H^+^-occluded state we performed crystallization experiments at acidic pH, and new LeuT crystal forms and structures were obtained (Table 1). Importantly, the structures show that an invariant Leu25 of the unwound region of TM1 is rotated and positioned into the substrate-binding pocket (Fig. 1a,b), while Glu290 remains occluded from the extracellular environment, presumably in a protonated state. Functional uptake and binding studies of human SERT with mutations of Leu99^hSERT^ (corresponding to Leu25^LeuT^; residue numbers in this paper will refer to the *A. aeolicus* LeuT sequence if not further specified) show that the uptake rates deteriorate even with highly conservative mutations, and combined with molecular dynamics (MD) simulations they suggest that the central Leu25 residue of TM1 plays a key role in the occlusion and gating of the inward-to-outward transition.

## Results

### LeuT crystal structure at Na^+^-free and low pH condition

We crystallized LeuT in a Na^+^-free, acidic pH environment to favour a protonated, substrate-free state. We obtained two crystal forms, which however display a closely related crystal packing that is also recurrent in other LeuT structures: a C2 form with one LeuT molecule in the asymmetric unit determined at 3.1 Å maximum resolution, and a P2_1_ form with two molecules and determined at 2.5 Å resolution (Table 1). The lower-resolution C2 form structure was obtained at pH 5.0 and overlays well with molecule B in the higher resolution P2_1_ form obtained at pH 6.5 (Supplementary Fig. 1a), for which the molecule A is slightly more open (Supplementary Fig. 1b). Therefore, the P2_1_ form is the main subject for further analysis as it shows both representations of the same functional state and is determined at a higher resolution.

**Figure 1:**
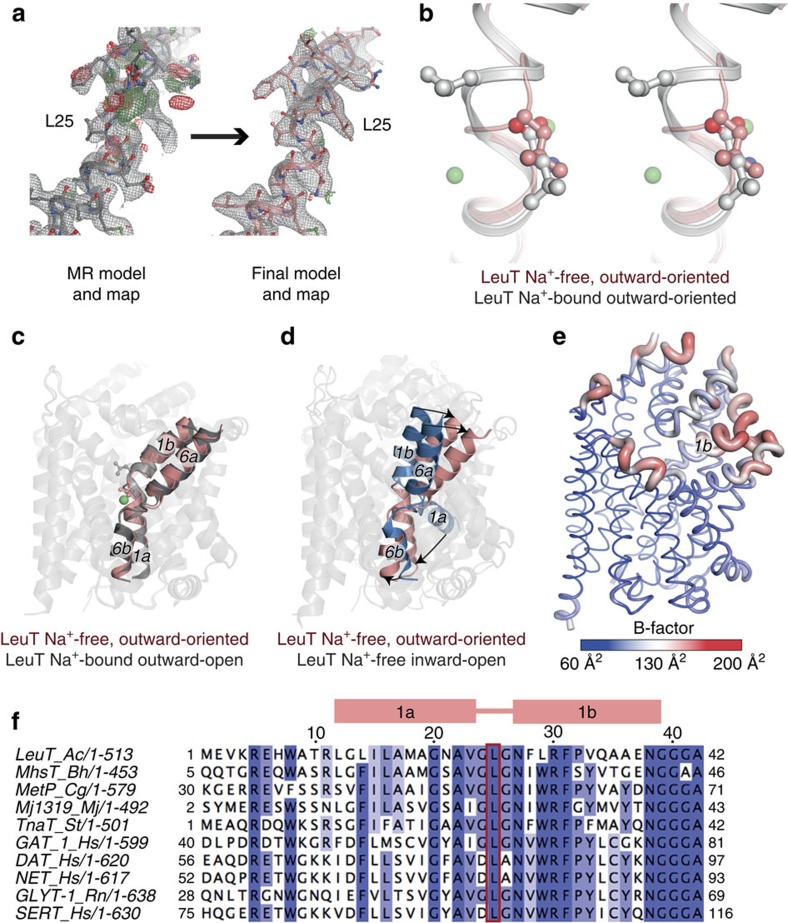
LeuT in the outward-oriented and Na^+^–free state shows relocation of conserved Leu25 into the substrate-binding site. (**a**) Electron density maps for TM1 after MR (left) showing unbiased indications for Leu25 relocation (final model and maps, right). 2F_o_-F_c_ maps are shown at 1 r.m.s.d. (grey mesh) and unbiased F_o_-F_c_ maps at 3.5 r.m.s.d. (green mesh positive, red mesh negative). (**b**) The outward-oriented, Na^+^-free state (pink, this study) superimposed on the Na^+^- and L-leucine-bound, occluded outward-oriented state^3^ (grey, PDB entry code 2A65). Leu25 overlaps with the substrate-binding site. (**c**,**d**) Outward-oriented Na^+^-free state (pink, this study) superimposed with Na^+^-bound outward-open (**c**, grey, PDB entry code 3TT1) and Na^+^-free inward-open (**d**, sky blue, PDB entry code 3TT3) states^2^. (**e**) Molecule A cartoon putty thickness illustrating flexibility and colour gradient for atomic displacement B-factor from blue (thin, low disorder) to red (thick, high disorder) with an average value of 93.4 Å^2^. (**f**) Alignment of the TM1 region of NSS. The conserved Leu25^LeuT^ is marked with a red box. MR, molecular replacement.

The overall structure of the Na^+^-free forms resembles the Na^+^-bound, outward-open^2^ or Trp-inhibited outward-facing states of LeuT^19^ that superimpose with 0.4–0.5 r.m.s.d. (Fig. 1c and Supplementary Fig. 1c). The intracellular side of the transporter is fully closed (Fig. 1c,d), while the extracellular side is slightly more open than for the outward-oriented, Na^+^/substrate-occluded state (Supplementary Fig. 1d). Thus, the extracellular cavity is exposed, and variations in the degree of opening (comparing molecules A and B) correlate with how *n*-octyl-β-D-glucoside (OG) molecules bind in that cavity at the Arg30-Asp404 gate (Supplementary Fig. 2a,b), similar to the outward-oriented, Na^+^/substrate-occluded state^20^. A highly flexible TM1b lines the extracellular cavity as indicated by relatively large B-factors of this region compared with the general structure (Fig. 1e) and as compared with the Na^+^-bound states^2^,^3^ (Supplementary Fig. 3).

**Figure 2:**
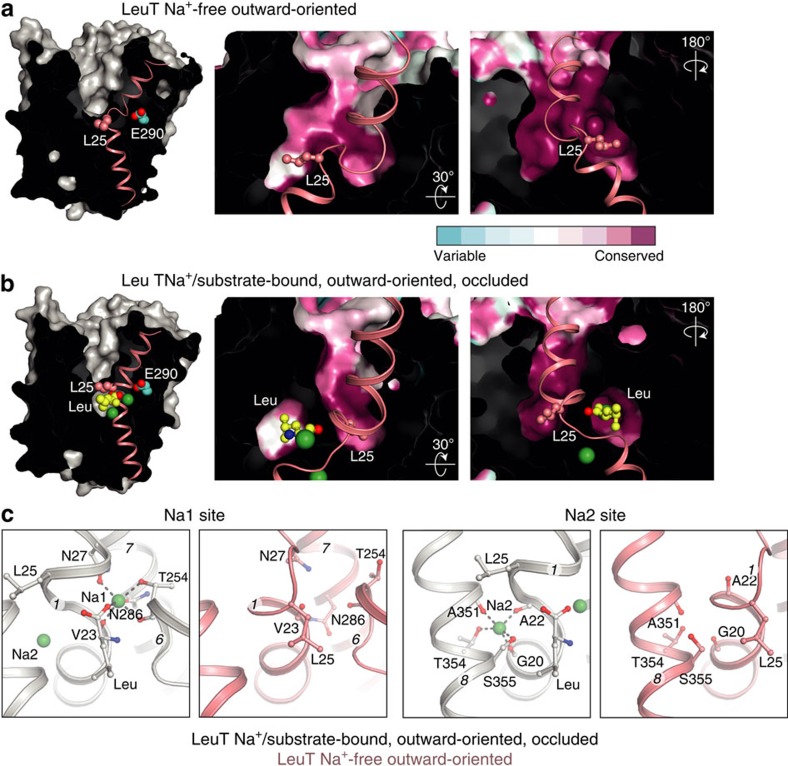
Leu25 and ion-binding sites. (**a**,**b**), The extracellular cavity and binding pockets for Leu25 and substrate in the Na^+^-free state (**a**, P2_1_ form molecule B, this study), and the Na^+^- and substrate-bound outward-occluded state^3^ (**b**, PDB entry code 2A65). The Na^+^ ions are shown as green spheres, Glu290 and L-leucine at the binding site by cyan and yellow spheres for carbon, respectively. The conservation is coloured from low (cyan) to high (magenta) using ConSurf^55^. (**c**) Na2 and Na1 sites in the outward-oriented Na^+^-free (pink, this study) and Na^+^-bound states with bound leucine substrate^3^ (grey, PDB entry code 2A65). Na^+^ binding and relocation of Leu25 to the substrate-binding site are mutually exclusive.

At the unwound part between TM1a and TM1b, we observe a key difference to the Na^+^-bound forms at the substrate-binding site: Leu25, which is conserved in all NSS (Fig. 1f), has rotated due to a neighbouring flip at the polypeptide chain, and it now directs the side chain into the vacant S1 substrate-binding site (Fig. 1b). The reorientation is facilitated by the two flanking glycine residues (Gly24 and Gly26, conserved in bacterial NSS and partially in mammalian members) (Fig. 1f) and it imposes an occlusion of Glu290 from the extracellular environment (Fig. 2a; see below). Different local changes would be expected for mammalian monoamine transporters that release a Cl^−^ ion instead of protonating a Glu290 side chain, and where the first glycine residue is replaced by an aspartic acid interacting with the amino group of the substrate. However, we anticipate that the rotation of the conserved Leu25 is a general NSS feature.

The residues that form the S1-binding pocket for the substrate amino and carboxyl groups are conserved among the NSS members, whereas the residues adjacent to the substrate side chain are far more variable (Fig. 2b). Similarly, the Leu25 residue occupies a conserved pocket formed by side chains of TMs 1, 3 6 and 8 in the Na^+^-bound states and inward-open apo state^2^,^3^. Notably, the reorientation observed here places the Leu25 side chain at the conserved part of the S1-binding site, that is, for the amino-acid moiety of the transported substrate (Fig. 2b). Thus, Leu25 has relocated from one to another conserved pocket.

### The Na^+^-free binding sites

**Figure 3:**
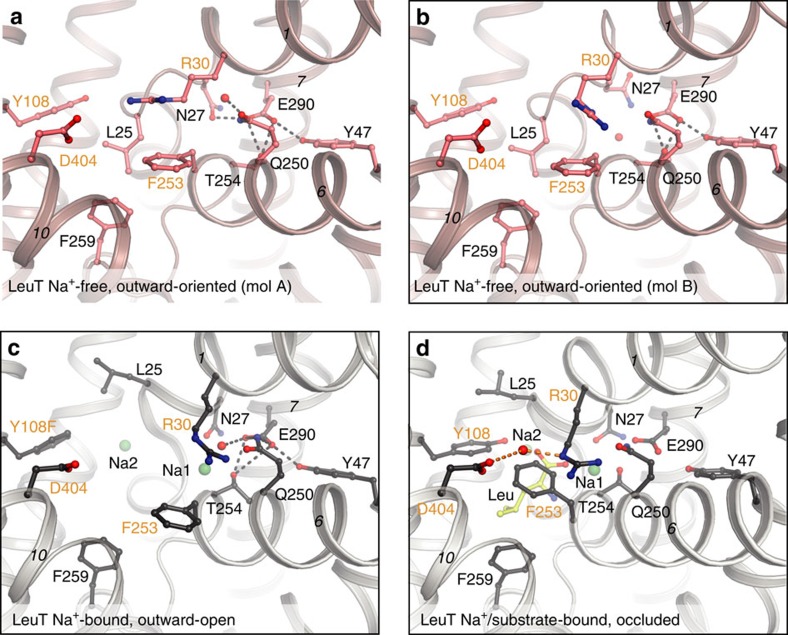
Extracellular gates and Glu290 interactions in different functional states of LeuT. For all panels a view from the extracellular side highlights the extracellular gates (orange labels) the Arg30-Asp404 salt bridge and the hydrophobic gate defined by Phe253 and Tyr108: (**a**) the outward-oriented, Na^+^-free state (molecule A, this study); (**b**) outward-oriented, Na^+^-free state (molecule B, this study) showing a different structure around Glu290; (**c**) Na^+^-bound, outward-open state^2^ (PDB entry code 3TT1); (**d**), Na^+^ and substrate-bound, outward-occluded state^3^ (PDB entry code 2A65). Glu290 is buried in the Na^+^-free state (**a**,**b**), which is therefore assumed to represent a neutral H^+^-occluded state of counter-transport.

The previously determined structure of LeuT in the inward-open apo state is also Na^+^-free, but stabilized by mutations at the Na2 site and an antibody^2^. Therefore, we paid particular attention to the Na^+^ sites in this the first Na^+^-free wild-type structure of a NSS protein. Indeed, no electron density is detectable for Na^+^ ions at the Na1 or Na2 sites. Rearrangement of side chains take place at the Na1 site, but somewhat surprisingly not at the Na2 site, which is the proposed driving site for all LeuT fold transporters^2^,^3^,^6^,^7^,^9^,^21^,^22^,^23^,^24^,^25^. The absence of Na^+^ ions at the Na1 and Na2 sites is in fact what also allows Leu25 to rotate on the basis of a local switch of the TM1 unwound region (Fig. 2c and Supplementary Fig. 4). Specifically, the carbonyl of Gly20 is rotated and the peptide bond between Val23 and Gly24 flipped thus at the same time distorting the Na2 site, which includes the Val23 carbonyl oxygen (Fig. 2c and Supplementary Fig. 4). For the Na1 site a rotation of the Ala22 carbonyl and the Asn27 side chain compensate for the absence of Na^+^ ion. The Asn27 and Thr254 side chains both interact with Na^+^ at Na1 in Na^+^-bound forms^2^,^3^, but now interact with the buried Glu290 side chain (molecule A; Fig. 3a,b). Na^+^ binding and the Na^+^-free conformation observed here therefore appear to be mutually exclusive, and Leu25 and Asn27 to play central roles in stabilization of a Na^+^-free form.

The Asn27 side chain shows variation in the two molecules in the asymmetric unit (Supplementary Fig. 2c,d and Supplementary Movie 1). In a slightly more open molecule A conformation (Supplementary Fig. 1b) Asn27 adopts a similar rotamer (Fig. 3a) as in the Na^+^-bound states^2^,^3^ (Fig. 3c,d); however, interacting directly with Glu290 side chain (Fig. 3a). In a more closed molecule B (similar to the C2 form at pH 5), Asn27 is moved away from Glu290 (Fig. 3b) and interacts with the backbone of Ala289 and Asn286 on TM7 (Supplementary Movie 1). However, we observe residual density in molecule A that superimposes with the molecule B rotamer of Asn27 and a water molecule, thus indicating flexibility of the structure in this region (Supplementary Fig. 2d) as also noted by relatively high B-factors (see above).

To our surprise, soaking of crystals with even 100 mM Na^+^ at pH 7.5 showed no indications of Na^+^ binding.

### LeuT Glu290 protonation

**Figure 4:**
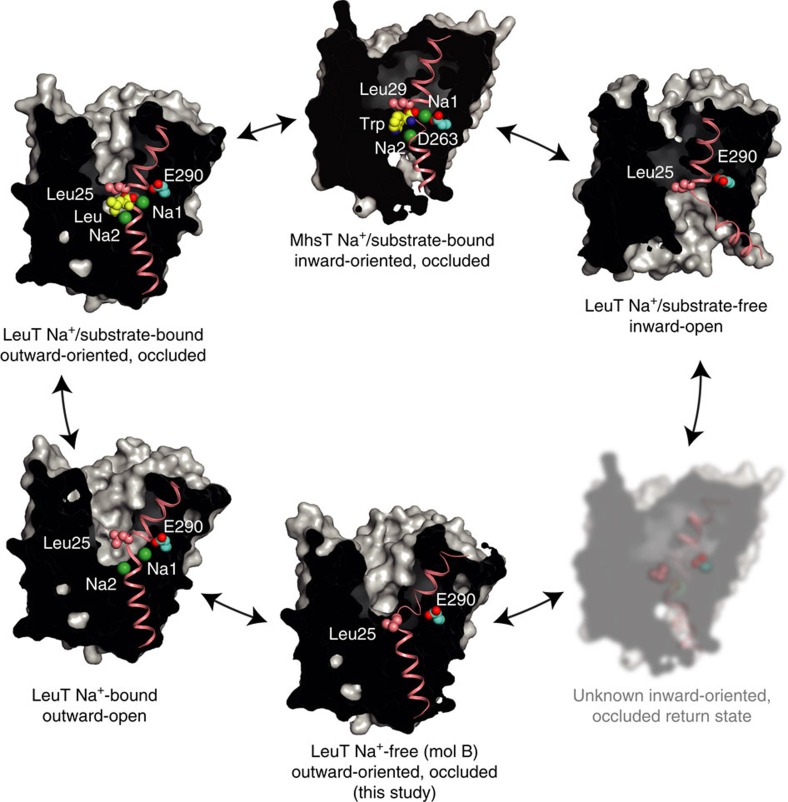
Comparison of the pathways and Glu290 accessibility in available LeuT and MhsT structures. LeuT outward-facing substrate-occluded state^3^ (PDB entry code 2A65); MhsT inward-facing substrate-occluded state^9^ (PDB entry code 4US3); LeuT inward-open apo state^2^ (PDB entry code 3TT3); LeuT Na^+^ free, outward-oriented return state; and LeuT outward-open Na^+^-bound state^2^ (PDB entry code 3TT1). The Glu290 residue (or for MhsT the proposed H^+^-binding residue Asp263) is buried in all of the states except outward-open, where it has a direct interaction with the extracellular environment. TM1 helix and Leu25 (Leu29^MhsT^) are shown in pink, the Na^+^ ions are shown as green spheres, Glu290 (or Asp263^MhsT^) as cyan and substrate at the binding site as yellow spheres.

Comparing all known LeuT structures, we note important differences in the solvent accessibility of Glu290, the proposed H^+^ antiport site (Fig. 4). Using PROPKA 3.1 (refs 26, 27), we also compared the estimated pK_a_ values of Glu290 (Supplementary Table 1), which were 5.4–6.2 for the Na^+^-bound, outward-facing states (Supplementary Table 1), that is, indicating that Glu290 is likely deprotonated in these states and in interaction with the positively charged Na^+^ ion at the Na1 site. In contrast, the pK_a_ value of Glu290 is estimated at 7.6 for the inward-open apo state, and 7.8–7.9 for the structures presented here (Supplementary Table 1). Considering the Leu25-mediated occlusion, the absence of Na^+^, and the close interaction of Asn27 and Thr254 to Glu290 (Fig. 3a,b), it suggests that our current structures represent an outward-oriented, H^+^-occluded state preceding ion exchange at the extracellular side, and thus functionally mirroring the inward-oriented Na^+^- and substrate-occluded state determined recently for MhsT^9^.

### Mutational studies of the conserved Leu25^LeuT^ in hSERT

Even though ligand-binding assays work well in LeuT^3^,^28^, these studies are performed under conditions that are very far from native conditions and LeuT is a very slow transporter under laboratory conditions. Furthermore, we wanted to study the effect of Leu25 mutations on the rate of the inward-to-outward-facing conformational change, which is further complicated by the fact that random insertion of LeuT into proteoliposomes leaves transporters with opposing orientations. We therefore turned to a well-established serotonin (5-HT) uptake system based on expression of hSERT in HEK293MSR cells^29^, which presents fully functional, uniformly oriented transporters in a near-native environment. Leu99^hSERT^, which is the equivalent residue to Leu25^LeuT^, was mutated to seven other hydrophobic residues (Ala, Val, Ile, Met, Phe, Tyr and Trp), and uptake of radiolabelled serotonin ([^3^H]-5-HT) was measured.

**Figure 5:**
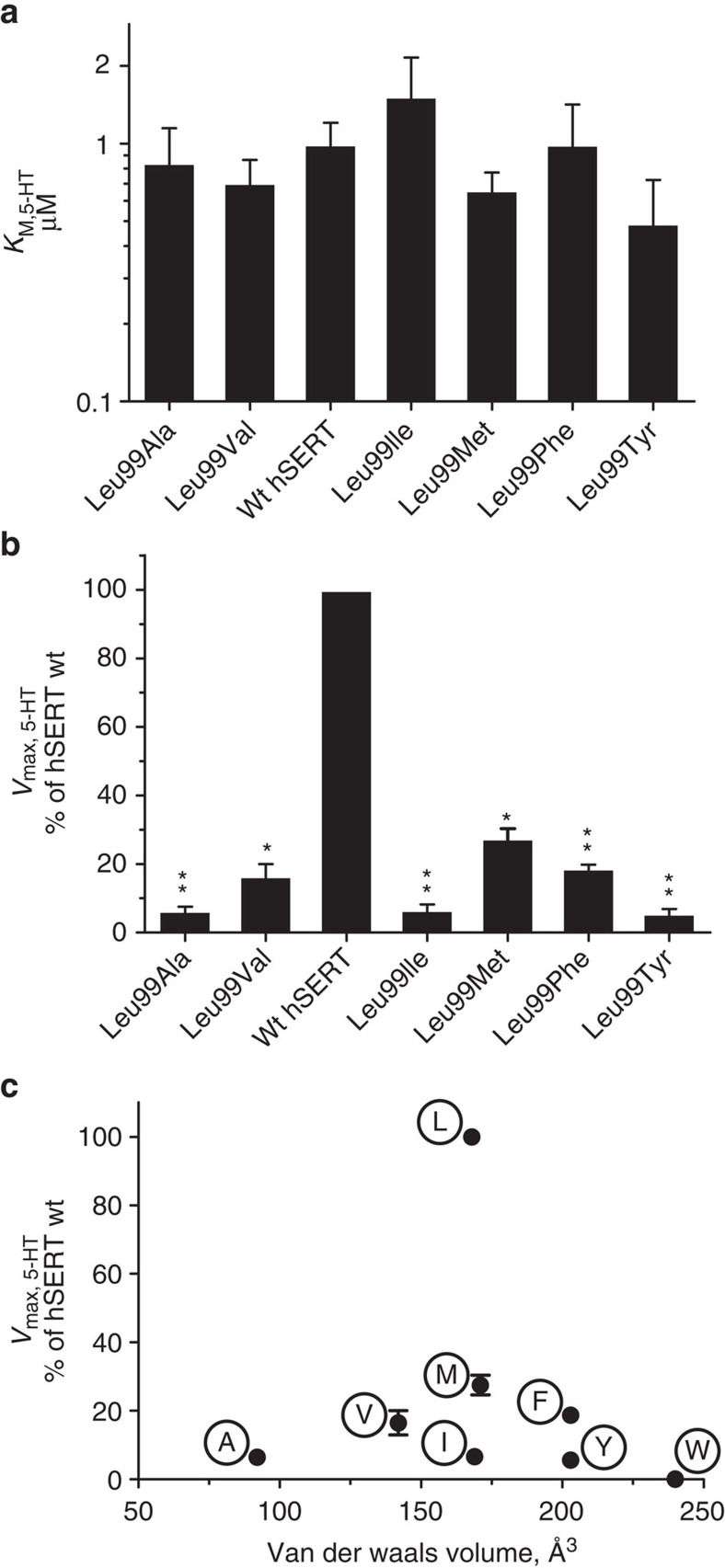
Leu99 in hSERT is crucial for the optimal transport rate. Leu99^hSERT^ is equivalent to Leu25^LeuT^ (see Fig. 1). (**a**,**b**), [^3^H]-5-HT uptake experiments in HEK-293-MSR cells expressing hSERT with Leu99 mutations show that the apparent substrate affinity is largely unaffected by mutations of Leu99 (**a**), whereas the maximum uptake rates are dramatically reduced for even the most conservative mutations (**b**,**c**), Transport rates show no systematic correlation with side chain volumes but rather a highly specific requirement for Leu, further supporting a specific two-site function of Leu99. For **a**–**c**, bars or points represent the mean from at least three independent experiments and the error bars the resulting s.e.m. A one-way ANOVA was used to compare *K*_M_ (**a**) or *V*_max_ (**b**) of each mutant to wt hSERT without any significant changes found for *K*_M_. All mutant *V*_max_ values were found to be significantly decreased relative to wt hSERT. *P<0.05, **P<0.01.

The Leu99Trp^hSERT^ mutation was completely inactive in transport, whereas mutations to other aromatic side chains (Phe and Tyr), smaller (Ala, Val), or similar size side chains (Ile and Met) were found to have *K*_M_ values similar to wild-type hSERT (0.8–1.5 times *K*_M_ for the wild type) (Fig. 5a). However, all active mutants, including the very conservative isoleucine mutation, exhibited a significantly decreased *V*_max_, ranging from 5 to 25% of the wild type (*P*<0.01) (Fig. 5b,c), hence demonstrating a very specific requirement for a leucine residue at this position for transport (Fig. 5c). These results are generally consistent with substrate binding being largely unaffected by a Leu99^hSERT^ mutation implicating that Leu99^hSERT^ is rotated out of the binding site before substrate binding. However, conformational transitions associated with the overall transport cycle are impaired when Leu99^hSERT^ is mutated even conservatively, that is, the rate-limiting inward-to-outward-facing transition is compromised by mutation of the Leu99^hSERT^ side chain. This could be due to either an unstable or an overly stabilized state of Na^+^-free mutant forms, where only a Leu99^hSERT^ side chain provides the balance for proper transport dynamics.

### Molecular dynamics simulations of Na^+^ binding

**Figure 6:**
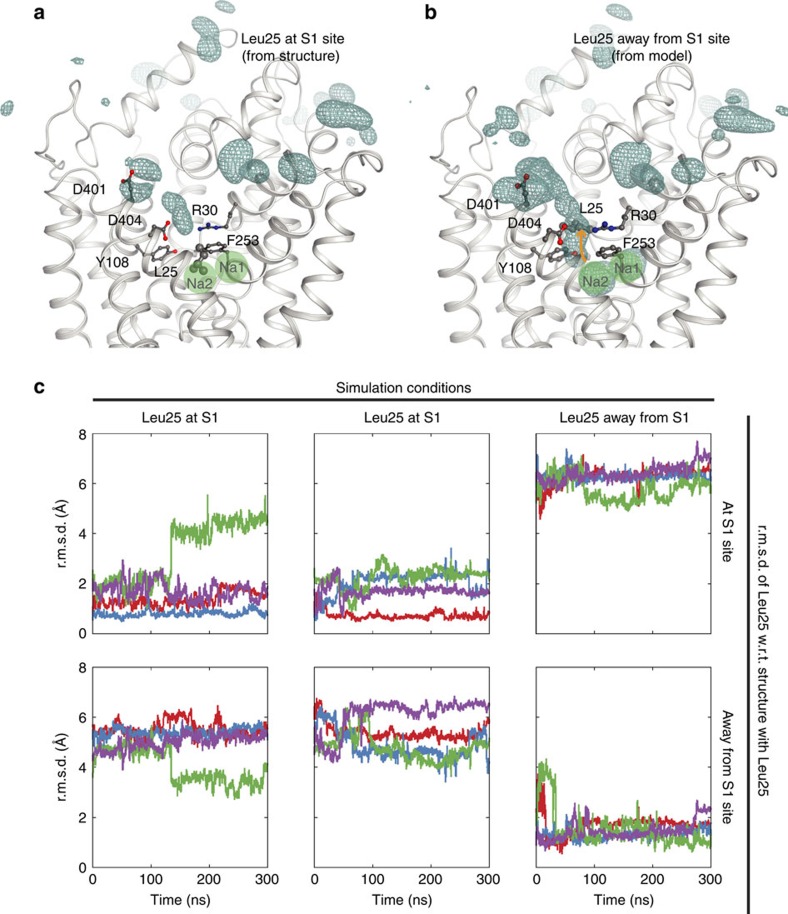
MD simulations of extracellular Na^+^ interactions in Na^+^/substrate-free, outward-oriented states. LeuT structure with Leu25 at S1 substrate-binding site (the crystal structure, (**a**) and with Leu25 rotated out of the substrate-binding site (modelled, **b**). The cyan mesh represents an isosurface corresponding to at least 1% Na^+^ occupancy of the volume during the simulations on the extracellular side. Green circles indicate the approximate positions of the Na1 and Na2 sites in **a**–**c**, r.m.s.d of Leu25 (calculated using the Cα, Cβ and Cγ atoms) for four independent MD simulations shown in different colours. The r.m.s.d is measured with respect to the outward-facing, Na^+^- and substrate-free return state of LeuT with Leu25 positioned at the S1 site (top) and with respect to the outward-facing, Na^+^- and substrate-bound state of LeuT with Leu25 positioned away from the S1 site (PDB entry code 2A65).

MD simulations were then performed for LeuT in the current form to analyse the stability of the observed conformation and the accessibility of the substrate- and ion-binding sites from the extracellular side. Four trajectories with a total simulation length of 1.2 μs were studied. During the simulations, the side chain of Leu25 remains stably positioned in the S1 substrate site, and no indications of Na^+^ ions interacting with the two Na^+^-binding sites were observed (Fig. 6a) regardless of whether Glu290 was protonated or not. However, Na^+^ ions do interact with residues in the extracellular vestibule, and in particular with the exposed and negatively charged residues Asp401 and Asp404. These two residues may serve to attract cations into the vestibule to facilitate subsequent binding of Na^+^ ions to the Na1 and Na2 sites. Yet, access is impaired by Leu25 occlusion indicating that conformational changes centred on Leu25 are required for Na^+^ binding to occur.

Qualifying this model, two 300 ns simulation on the LeuT dimer (Glu290 residues not protonated) were performed with residues 24–26 assuming a conformation that resembles the Na^+^-bound outward-occluded form^3^ (Protein Data Bank (PDB) entry code 2A65). In all of four monomer trajectories, Na^+^ is now observed to enter the central binding sites, and in two of them a Na^+^ ion eventually binds at the Na1 site, while stable binding at the Na2 site is not observed (Fig. 6b). Thus, our simulations point to a crucial role of Leu25 as a gatekeeper for Na^+^ binding to the outward-facing Na^+^-free form.

## Discussion

Our structures of LeuT in an outward-oriented, Na^+^-free state provide new insights into the mechanism of counter-transport and return transition of the NSS transporters for renewed binding of Na^+^ and substrate from the extracellular side. The structures pinpoint Leu25 of LeuT—a completely conserved residue in the NSS family—as a key residue for stabilization of the empty substrate and Na^+^ sites associated with the inward-to-outward conformational transition. This was also supported by mutational studies of the equivalent Leu99 in hSERT, where even the most conservative mutations of this residue had profound effects on maximal transport rates, but not on apparent substrate affinity. These findings point to a crucial role for hSERT Leu99 taking part in a rate-limiting step^18^, which does not interfere with substrate binding *per se*. It is consistent with the LeuT structure and MD simulations we present here that identify the equivalent Leu25 as an important gatekeeper of the Na^+^-free return state. Similar substrate-binding site compensation is seen for the glutamate transporter family (by a conserved arginine) with a completely different structure and topology^30^,^31^. On the other hand, the betaine transporter BetP, which has the same topology as LeuT, does not show a similar side chain rotation in substrate-free states^24^.

Importantly, the current structures show a buried Glu290 with no positive charges to compensate a negatively charged carboxylate (as opposed to the Na^+^-bound states) and an increased predicted pK_A_ indicating that Glu290 is protonated and neutral. Presumably our LeuT structure therefore represents an occluded return step of H^+^ counter-transport that from a functional point of view would correspond to a K^+^-occluded state of SERT. Deprotonation of Glu290 and a disengagement of Leu25 from the substrate-binding pocket are likely to be coupled events that precede Na^+^-binding on the extracellular side. In support of a coupled mechanism, a recently published MD study^32^ suggested that deprotonated Glu290 attracts Na^+^ towards the Na1 site for the outward-facing state of LeuT, while protonation of Glu290 in the inward-facing state stimulates a release of Na^+^ from the Na1 site. Indeed, mutational studies implicate the Na1 site in modulation of conformational changes associated with Na^+^ binding that are otherwise primarily driven by the Na2 site^33^. These effects may reflect the preparatory steps of Na^+^ binding at Na1 described here.

A general flexibility of TM1b, and specifically in interactions of Asn27 with the buried Glu290 side chain, is observed by morphing between the two protomers of our two crystal forms (Supplementary Movie 1). The flexibility may play a role in facilitating H^+^ release, similar to TM5 flexibility facilitating Na^+^ release to the intracellular environment from the inward-oriented, substrate-occluded state^9^. Glu290 deprotonation might further increase the flexibility of TM1b and in itself facilitate the rotation of Leu25 out of the substrate-binding site (and hence Na^+^ binding), but we see no immediate support for this in our simulations (Fig. 6). However, a reverse H^+^ gradient (high pH outside) stimulates transport of bacterial NSS^15^. Thus, flexibility of the outward-oriented form of the return transition may allow H^+^ release (or K^+^ release for SERT) and accelerate the exposure of Na^+^ sites (and transient pre-sites) to the high Na^+^ concentration of the extracellular environment.

Earlier MD simulations have suggested that extracellular Na^+^ binds first at the Na2 site of LeuT, which then increases the affinity for Na^+^ at the Na1 site^34^. However, we see no immediate binding of Na^+^ in simulations starting from our structure, but only when Leu25 rotation is manually introduced, and then first at the Na1 site. Our findings are therefore more consistent with a recent study by Zomot *et al*.^35^, where simulations were started from an outward-oriented conformation of LeuT with ions modelled out (and including also Leu25 in the Na^+^-bound conformation). Here, Na^+^ binding was also observed to occur first at the Na1 site rather than the Na2 site. However importantly, our MD simulations show that Leu25 must move out of the S1 site first (Fig. 6a) accompanied by a flip of the Val23 carbonyl to prepare the Na1 and Na2 sites for Na^+^ binding (Fig. 6b). Na^+^ binding is not compatible with the S1 location of Leu25, so we identify Leu25 as a gatekeeper for extracellular Na^+^ binding in the outward-facing return state that blocks Na^+^ influx with inward-outward return transition dynamics. Leu25 complements the Arg30-Asp404 gate, which in the current H^+^-occluded state is only semi-closed. In Na^+^ bound forms Arg30-Asp404 on the other hand becomes a defining gate that closes when substrate binds. In other words, Leu25 location to S1 appears to play the same role for Na^+^-free occlusion in the inward-outward return transition as the S1-bound substrate has for Na^+^-bound occlusion in the outward-inward uptake transition^2^,^3^.

The existence of a transient Na^+^-site adjacent to Na1, coined Na1', has been inferred from the MD simulations of Na^+^ binding^32^,^35^,^36^,^37^. We observe no similar Na1' site in our simulations, only a more peripheral transient site in the extracellular vestibule at Asp401 and Asp404, and also no Na^+^ binding in soaked crystals. Na^+^ binding will obviously depend on the local structure of the TM1a-TM1b connecting segment, which has so far only been modelled similar to Na^+^-bound forms in simulations of Na^+^-binding. The Na1' site may indeed emerge as a transient holding position for Na^+^ that is sensitive to the protonation state of Glu290 (ref. 32), but importantly our results (by 300 ns simulations and crystal soaking) indicate that Leu25 rotation out of the S1 substrate site must precede for Na^+^ interactions to occur.

MD simulations, mutational studies and single-molecule fluorescence resonance-energy transfer data have also implicated a second substrate site (S2) in the LeuT transport cycle^28^,^38^, but so far concerning the mechanism of Na^+^ and substrate release in the outward-to-inward transition. We see no direct implication of the Leu25 switch in the S2 functionality, although we observe the Phe320 residue that proposedly is part of the S2 site^28^ to adopt different local structures in the two copies of the P21 crystal form, and we cannot exclude that the S2 site may play a role in stimulating, for example, proton release and Leu25 relocation.

**Figure 7:**
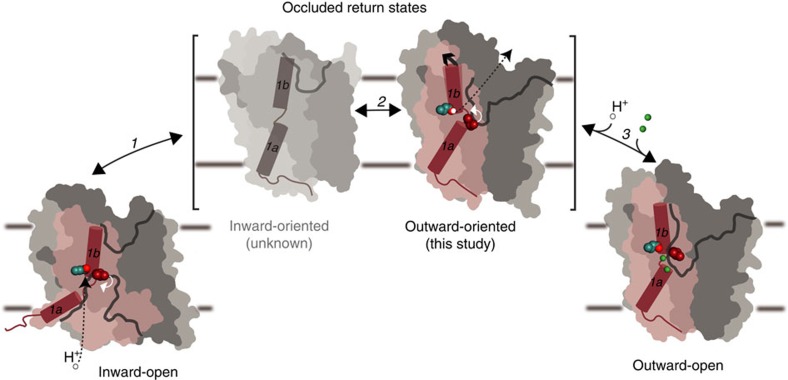
Inward-to-outward return transition of the NSS family. In the inward-open state, LeuT gets protonated on Glu290 (ref. 15), and Leu25 (red spheres) presumably moves into the substrate-binding pocket (white arrow) (1). From an occluded inward-oriented return state LeuT can switch to the occluded outward-oriented state described here (2). TM1b is flexible and this state is likely prone to H^+^ release (dashed line) and Leu25 relocation out of the substrate-binding pocket (white arrow) allowing Na^+^ ions to bind (3). Na^+^ binding at the Na1 and Na2 sites stabilize the outward-open state and the transporter is ready for substrate binding and the forward transport cycle.

With new LeuT structures depicting an outward-oriented, H^+^-occluded state, we propose the following rationales of the return steps of the transport cycle of the NSS family (Fig. 7; full transport cycle addressed in the Supplementary Discussion, Supplementary Fig. 5 and Supplementary Movie 2): (1) Upon substrate and Na^+^ release in the inward-open state the Glu290^LeuT^ residue (or equivalent residues in bacterial NSS) is protonated, or a K^+^ counter-ion is bound (for example, for SERT), leading to closure of the intracellular pathway. We propose that Leu25^LeuT^ (or the equivalent leucine residue in other NSS) already here rotates into the S1 substrate-binding site and stabilizes a Na^+^- and substrate-free, inward-oriented and occluded structure (which, however, is still unknown for NSS). Dynamics of the occluded transporter now allows it to also explore the outward-oriented, occluded state described here, but Leu25 gating ensures that extracellular Na^+^ binding and influx will not take place. (2) In the outward-oriented state flexibility of TM1b at the extracellular vestibule facilitates the release of a counter-transported ion (if present), that is, H^+^ or K^+^, to the extracellular environment and the disengagement of Leu25^LeuT^ from the S1 substrate site (Fig. 1e). (3) Na^+^-binding sites are now able to interact with the extracellular, high Na^+^ concentration environment, and the transporter becomes primed for substrate binding and a forward transport cycle. Overall, this dual role of Leu25 of first compensating and stabilizing substrate- and Na^+^-free sites to facilitate the inward-to-outward-facing return transition, followed by rotation out of the substrate site to gate Na^+^ binding from the extracellular milieu ensures vectorial control in the return transition against a Na^+^ gradient.

Further investigations are clearly required to derive a complete mechanism of the coupled processes of counter-transport, Leu25 rotation and Na^+^ and substrate binding in NSS transporters. Yet, together with inward- and outward-oriented structures of the Na^+^- and substrate-occluded states of NSS protein, the current LeuT structures point in concrete terms to how an outward-to-inwardly directed Na^+^ gradient, together with extracellular substrate, stimulates a secondary transporter to assume net forward transport and substrate accumulation in coupled symport with Na^+^.

## Methods

### LeuT purification

The *leuT* gene flanked by a sequence that encodes a C-terminal 6 × His tag and thrombin protease cleavage site (in plasmid pET16b) was expressed in *Escherichia coli* C41 (DE3) cells, re-suspended in ice-cold buffer A (50 mM Tris-HCl pH 8, 200 mM KCl, 20% glycerol). Phenylmethanesulfonylfluoride (PMSF, Sigma) was added to the re-suspended cells to a final concentration of 1 mM and cells were disrupted using a high-pressure homogenizer (Avanti HPH) by three passes at 15,000 psi. Opened cells were centrifuged at 23,665*g* for 20 min at 4 °C to remove debris and non-broken cells. The supernatant was transferred to fresh tubes and membranes were collected by ultracentrifugation at 243,500*g* for 2 h at 4 °C. Membranes were re-suspended in ice-cold buffer A (1 ml for 1 g of wet membranes). For solubilization of the LeuT, membranes were incubated with 40 mM *n*-dodecyl-β-D-maltoside (DDM SOL-GRADE, Affymetrix) for 1 h at 4 °C in a beaker while stirring and then proceeded to Ni^2+^ affinity chromatography. The solubilized membranes were incubated with Ni-NTA resin (QIAGEN, 1 ml per 10 mg of protein) pre-equilibrated in buffer B (50 mM Tris-HCl pH 8, 200 mM NaCl, 20% glycerol, 10 μM L-leucine, 1 mM) and supplemented with 50 mM imidazole to avoid non-specific binding. The protein loaded Ni-NTA resin was packed into a C column (GE Healthcare), connected to the Äkta purifier system (GE Healthcare), and was washed with buffer B supplemented with 50 mM imidazole to remove any residual contaminants. The protein was eluted in a one-step elution with buffer B supplemented with 300 mM imidazole. The mutant T354H was prepared by site-directed mutagenesis and purified as WT in Na^+^-free conditions.

### Crystallization and data collection

LeuT WT and T354H mutant were purified in *n*-dodecyl-β-D-maltoside (DDM, SOL-GRADE, Anatrace) using His_6_-tag chromatography (Ni-NTA, Qiagen). The eluate was digested with thrombin protease (Calbiochem) to remove the His-tag and passed again over the Ni-column where the LeuT-containing flow-through was collected. The protein was concentrated using a Viva-spin concentration device with molecular weight cutoff of 50 kDa to 4 mg ml^−1^ for size exclusion chromatography on a 15 ml KW803 silica column (Shodex) with a running buffer consisting of 10 mM Tris-MES pH 6.0, 100 mM KCl, 10% (v/v) glycerol, 40 mM *n*-octyl-β-D-glucoside (2 × CMC; ANAGRADE, Anatrace). Peak fractions were collected and concentrated to 3–4 mg ml^−1^ using a Viva-spin concentration device with a molecular weight cutoff of 50 kDa.

Reservoir buffer containing 100 mM Tris-MES pH 5.0, 100 mM KCl, 20–24% w/w PEG550 MME and 10% (volume) glycerol, or 100 mM Tris-MES pH 6.5, 75 mM K-citrate, 24–28% w/w PEG550 MME and 10% (volume) glycerol, or for the T354H mutant 100 mM Tris-MES pH 5.5, 100 mM KCl, 20–24% w/w PEG550 MME and 10% (volume) glycerol, was mixed with the protein solution as 1+1 μl drops and subjected to vapour diffusion at 19 °C in sitting drops. For cryo-protection the reservoir, PEG550 MME concentration was increased to 35% and the drops left to equilibrate overnight. The crystals were mounted in litholoops (Molecular Dimensions, UK) and flash cooled in liquid N_2_. X-ray diffraction data were collected at the DLS I24 microfocus beamline and processed and scaled in space group C2 for the WT pH 6.5 and T354H form, and P2_1_ for the WT 5.0 form, respectively using the X-ray diffraction package^39^ and TRUNCATE^40^ yielding final data sets at 2.5, 2.6 and 3.1 Å maximum resolution, as judged from the Wilson plot, the CC-0.5 coefficient, and model refinement statistics (see Table 1). The quality of the data sets was further evaluated with PHENIX XTRIAGE^41^, and for anisotropy by the Diffraction Anisotropy Server^42^, which indicated a severe anisotropy for the C2 form, for which anisotropy correction hence was applied (Table 1).

### Structure determination and analysis

Initial phases were obtained by molecular replacement with PHENIX AUTOMR^41^ using the wild-type LeuT-Trp^19^ (PDB entry code 3F3A) outward-open structure with removed ligands as the search model. Iterative rounds of structure refinement were performed in PHENIX REFINE^41^ with model rebuilding in COOT^43^. The final refinement statistics are summarized in Table 1 (PDB entry codes 5JAE, 5JAF and 5JAG).

Figures were created using PYMOL^44^. The movies (Supplementary Movie S1 and S2) were generated as a morph between structures using PYMOL^44^ with geometry minimization for each iteration performed using PHENIX^41^. A multiple sequence alignment of the NSS family was created using Muscle^45^. The pK_a_ calculations were done with PROPKA 3.1 (refs 26, 46, 47).

### 5-HT uptake assays in cell culture

Mutagenesis of hSERT cDNA in the pcDNA3 vector (Invitrogen) was carried out using Phusion High-Fidelity DNA Polymerase (Finnzymes), and transformed in XL10 Gold *Escherischia coli* (Stratagene) competent cells. The mutation was confirmed by sequencing across the entire reading frame.

The uptake measurements were performed as previously described^29^. HEK-293 MSR cells (Invitrogen) were cultured as monolayer cultures in DMEM (BioWhitaker) supplemented with 10% FCS (Gibco Life Technologies), 100 U ml^−1^ penicillin, 100 μg ml^−1^ streptomycin (BioWhitaker) and 6 μg ml^−1^ of Geneticin (Invitrogen) at 95% humidity and 5% CO_2_ at 37 °C. Two days before the uptake experiment, cells were detached from the culture flask with trypsin/EDTA (BioWhitaker), transfected with midiprep DNA-Lipofectamine 2000 (Life Technologies) complex and seeded into white tissue culture treated 96-well microtiter plates (Nunc). Immediately before the uptake experiment was initiated, medium was aspirated and cells were washed once with PBSCM (137 mM NaCl, 27 mM KCl, 4.7 mM Na_2_HPO_4_, 1.2 mM KH_2_PO_4_, 0.1 mM CaCl_2_, 1 mM MgCl_2_, pH 7.4). Cells for determination of non-specific uptake were preincubated with 200 μM imipramine for 30 min, while cells for determination of total uptake were incubated with PBSCM. Uptake was initiated by the addition of 40 μl of a dilution of the [^3^H]-5-HT mixed with unlabelled 5-HT in a 1:10 ratio. Uptake was terminated after 10 min by aspiration and washing with PBSCM. All wash steps were done on a Bio-Tek Instruments ELx50 automatic strip washer. 50 μl of Microscint 20 (Packard) was dispensed into each well resulting in cell lysis and release of accumulated radiolabelled substrate from the adherent cells allowing direct quantitation on a Packard topcounter. Uptake data were fitted to Michaelis–Menten kinetics by nonlinear regression analysis using the built-in tools in Prism5 (Graphpad).

### MD simulations

The simulation system for the MD simulations was based on a crystal structure of a T354H mutant adopting the same structure as described here for the wild-type structure obtained at pH 5.0 and molecule A at 6.5, but early on determined at 2.6 Å resolution and therefore available at higher resolution than for the wild-type structure at that time (3.2 Å). Residue His354 was mutated *in silico* back to threonine and the side chain positioned according to the side chain position of Thr354 in the lower-resolution wild-type structure. We later found no significant differences to the 2.5 Å resolution wild-type structure, in particular taking the MD preparatory steps into account (see next), and simulations therefore were not rerun. The structure was prepared for MD simulations using the Protein Preparation Wizard Epik version 2.3 and Prime version 3.1 in the Schrödinger Suite 2012 (Schrödinger, LLC). The detergent molecules in the crystal structure were deleted. The pK_a_ values for all titratable groups were estimated using PROPKA 3.1 (refs 26, 46, 47). On the basis of these results and the local environments with respect to hydrogen bonding, Glu112, Glu287, Glu290, Glu368 and Glu419 were all modelled as protonated, while the remaining aspartate and glutamate residues were modelled as charged. Furthermore, His74 was modelled as charged. His377 and His480 were modelled as the ɛ-tautomer, and the remaining His residues were modelled as δ-tautomers. The side chain of Lys288, which is positioned towards the hydrophobic part of the membrane, was modelled as neutral. In addition, two other setups were constructed, one in which Glu290 was modelled as charged, and one in which the residues 24–26 were modelled as in the substrate-bound state seen in PDB 2A65 such that the side chain of Leu25 is not pointing into the central binding site. For each setup, the following steps were performed in VMD version 1.9.1 (ref. 48). The observed LeuT crystal dimer was inserted into a lipid bilayer consisting of 1-palmitoyl-2-oleoyl-*sn*-glycero-3-phosphoethanolamine (POPE) lipids. The size of the membrane was chosen such that at least 20 Å of lipids surrounded the dimer. The dimer was positioned in the membrane according to the alignment of pdb structure 3TT1 in the Orientations of Proteins in Membranes database^49^. The system was solvated by adding a 15 Å layer of water molecules on both sides of the membrane. NaCl was added to a concentration of 0.2 M. The simulation system contained ∼130,000 atoms and the equilibrated system had a box size of ∼125 × 100 × 105 Å^3^. The simulations were performed in Gromacs version 4.6.3 (ref. 50) using the CHARMM36 force field^51^,^52^ for the protein and lipids and TIPS3P water parameters^53^,^54^. Bond lengths were kept fixed used the LINCS algorithm, enabling the use of a 2 fs time step. Coordinates were tracked in 5 ps intervals. The temperature was kept at 310 K using a Nosé-Hoover scheme and the pressure was maintained at 1 atm using a Parrinello-Rahman approach with semi-isotropic scaling. A cutoff at 12 Å was applied for the van der Waals interactions using a switch function starting at 10 Å. The cutoff for the short-range electrostatic interactions was at 12 Å and long-range electrostatic interactions were calculated using Particle-Mesh Ewald. Two simulations with different initial velocites were performed using the following approach for each setup: A steepest descent minimization of 10,000 steps of the entire simulation system was performed followed by a 0.5 ns MD simulation in the NVT ensemble at 310 K with restraints on all atoms (*k*=1,000 kJ mol^−1^ nm^−2^) except the lipid tails. The system was further equilibrated in the NPT ensemble through a 2 ns MD simulation with positional restraints on the protein (*k*=1,000 kJ mol^−1^ nm^−2^) followed by a 4 ns equilibrium MD simulation. Finally a production MD simulation with a simulation time of 300 ns was performed. As each simulation setup includes a LeuT dimer, this approach leads to two LeuT monomer trajectories per simulation, and a combined trajectory lenght of 1.2 μs for analysis for each of the three setups.

## Additional information

**Accession codes:** Coordinates and structure factors have been deposited in the Protein Data Bank under accession code 5JAE (P2_1_ form, pH 6.5), 5JAF (C2 form, pH 5) and 5JAG (C2 form, T354H mutant, pH 6).

**How to cite this article:** Malinauskaite, L. *et al*. A conserved leucine occupies the empty substrate site of LeuT in the Na^+^-free return state. *Nat. Commun.* 7:11673 doi: 10.1038/ncomms11673 (2016).

## Supplementary Material

Supplementary Information Supplementary Figures 1-5, Supplementary Table 1, Supplementary Discussion and Supplementary References

Supplementary Movie 1 The movie depicts the transition between two LeuT return states in pH 6.5 structure Molecule A and B, zoomed in at the extracellular side. The movie was prepared by 'morphing' from Molecule A to Molecule B and back. TM1b (red cartoon) fluctuates to some extent at the extracellular side, however Leu25 (red sticks) is stable at its position. The major difference between the two molecules is Asn27 (orange sticks), which interacts with either the Glu290 side chain (yellow sticks) or the TM7 backbone next to Glu290

Supplementary Movie 2 The movie depicts the full transport cycle of the NSS family going from the outward-facing return state (presented here) through all known states of the NSS cycle (see supplementary discussion and supplementary figure 6 for further details). The movie 'morphs' between available structures - LeuT outward-facing, Na+-free return state (this study), LeuT outward-open, Na+-bound1 (PDB entry code 3TT1), LeuT outward-oriented substrate-occluded (PDB entry code 2A65), MhsT inward-oriented and substrate-occluded (PDB entry code 4US3), and LeuT inward-open apoform (PDB entry code 3TT3). For compatibility in morphing procedures, a leucine and sodium-bound inward-oriented and occluded state was modeled for LeuT based on the MhsT structure as described in Methods. The structures were aligned on the scaffold domain (TMs 3-4, 8-9). TM5 is shown as a cyan helix, TM1 as red helix, Na+ ions at Na1 and Na2 sites are shown in green, leucine in orange.

## Figures and Tables

**Table 1:** Data processing and refinement statistics.

**Data set**	**pH 6.5**	**pH 5.0**	**T354H mutant**
PDB	5JAE	5JAF	5JAG
Beamline	DLS I24	DLS I24	DLS I24
Wavelength (Å)	0.9686	0.9686	0.9686
Space group	P2_1_	C2	C2
No. crystals	1	1	1
Unit cell parameters (Å, °)	81.6, 92.1, 92.5, 90, 95.2, 90	86.0 88.9 80.990.0 94.3 90.0	88.2, 95.6, 83.7, 90, 97.8, 90
Mosaicity (°)	0.08	0.27	0.14
Resolution (Å) (outer bin)	49.3–2.50 (2.59–2.50)	30.0–3.08 (3.16–3.08)	36.5–2.58 (2.69–2.58)
Wilson B-factor	68.4	94.0	63.9
*I*/*σI*	12.1 (0.9)	9.2 (0.9)	10.7 (1.0)
*I*/*σI*^aniso corr^		13.0 (3.3)	
*R*_meas_ (%)	2.3 (223.9)	12.4 (173)	7.6 (95.0)
*R*_meas_ (%)^aniso corr^		7.5 (36.8)	
*R*_pim_ (%)	3.7 (82.9)	6.7 (91.9)	4.9 (60.7)
CC_1/2_	99.9 (49.6)	99.8 (63.6)	99.9 (68.7)
Completeness (%)Completeness (%)^aniso corr^	99.1 (98.5)	99.6 (99.7)75.1 (5.1)	97.4 (98.8)
Redundancy Redundancy^aniso corr^	6.9 (6.8)	3.4 (3.5)2.5 (0.2)	2.2 (2.1)
Residues and ligands	1,010 Residues11 OG, 44 waters	494 Residues4 OG	496 Residues7 OG, 9 waters
*R*/*R*_free_*R*/*R*_free_^aniso corr^	0.218/0.247	0.243/0.295 0.207/0.251	0.203/0.240
Average B-factor (Å^2^)			
Overall	94.3	65.0	76.7
Waters	83.9	—	59.5
Detergents	114.1	90.7	125.6
r.m.s.d. bond angles (°)	0.701	0.758	0.849
r.m.s.d. bond lengths (Å)	0.003	0.003	0.004
Ramachandran plot favoured/outliers (%)	97.5/0.1	96.9/0.0	97.6/0.0

OG, *n*-octyl-β-D-glucoside.
