# Supplementary material for: A conserved leucine occupies the empty substrate site of LeuT in the Na+-free return state
Source: Nat Commun. 2016 May 25;7:11673. doi: 10.1038/ncomms11673 (PMC4894957; doi:10.1038/ncomms11673)
Supplement: Supplementary Information — Supplementary Figures 1-5, Supplementary Table 1, Supplementary Discussion and Supplementary References [file ncomms11673-s1.pdf]

Supplementary Figures

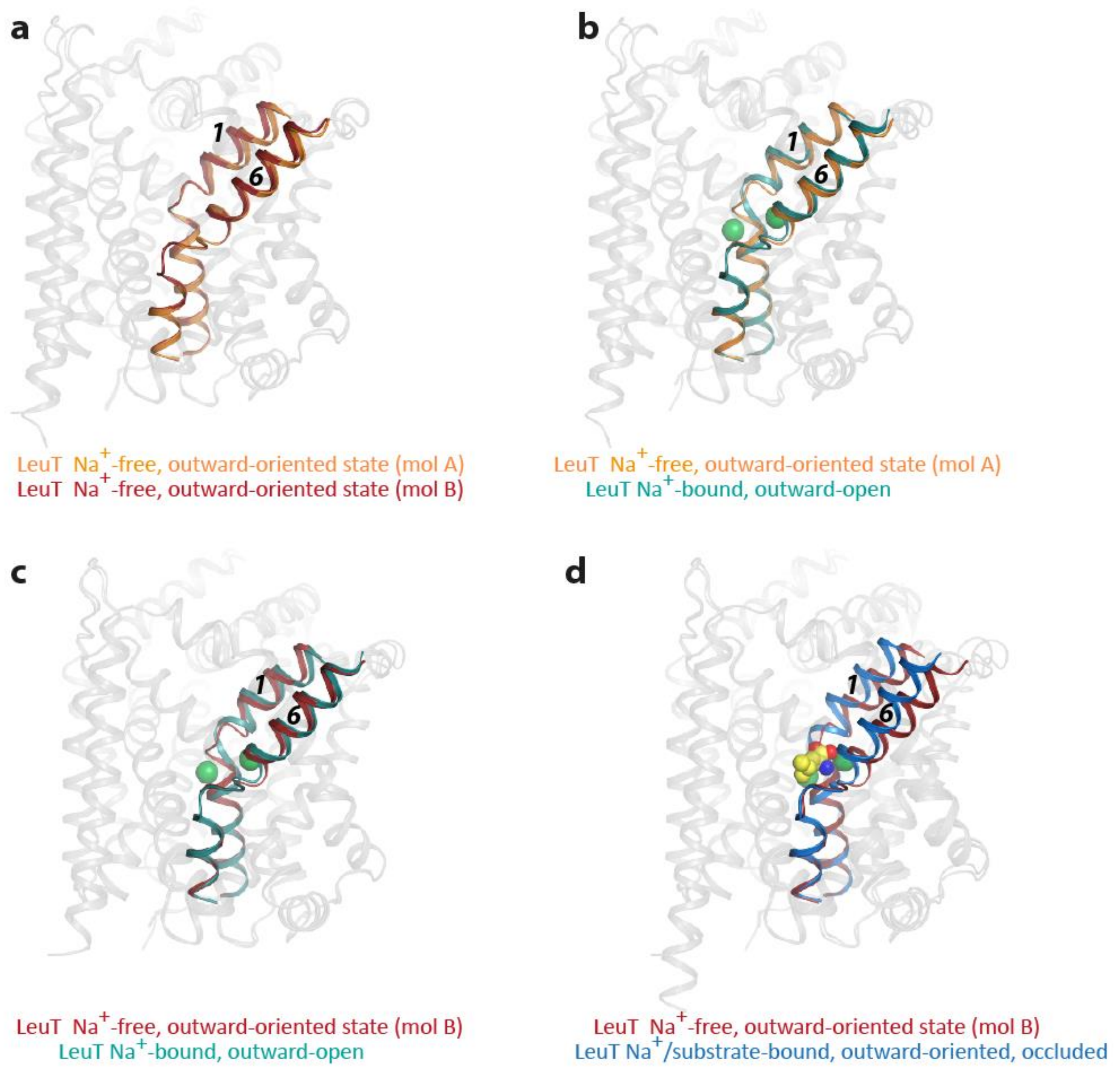

Supplementary Figure 1. LeuT return outward-open structures overlay with available outward-oriented LeuT structures: a, LeuT return state pH 5.0 (yellow) and and Molecule B (red); **b**, LeuT pH 6.5 Molecule A (orange) and Molecule B (red); **c**, LeuT pH 6.5 Molecule A and outward-open Na^+-bound state[1] (cyan, PDB entry code 3TT1); **d**, LeuT pH 6.5 return state Molecule B and LeuT outward-occluded substrates-bound state[2] (blue, PDB entry code 2A65). The major differences are seen in the positions of TM1 and TM6 (showed as colored helices). Na^+ ions are shown as green spheres and leucine at the binding site as yellow spheres.

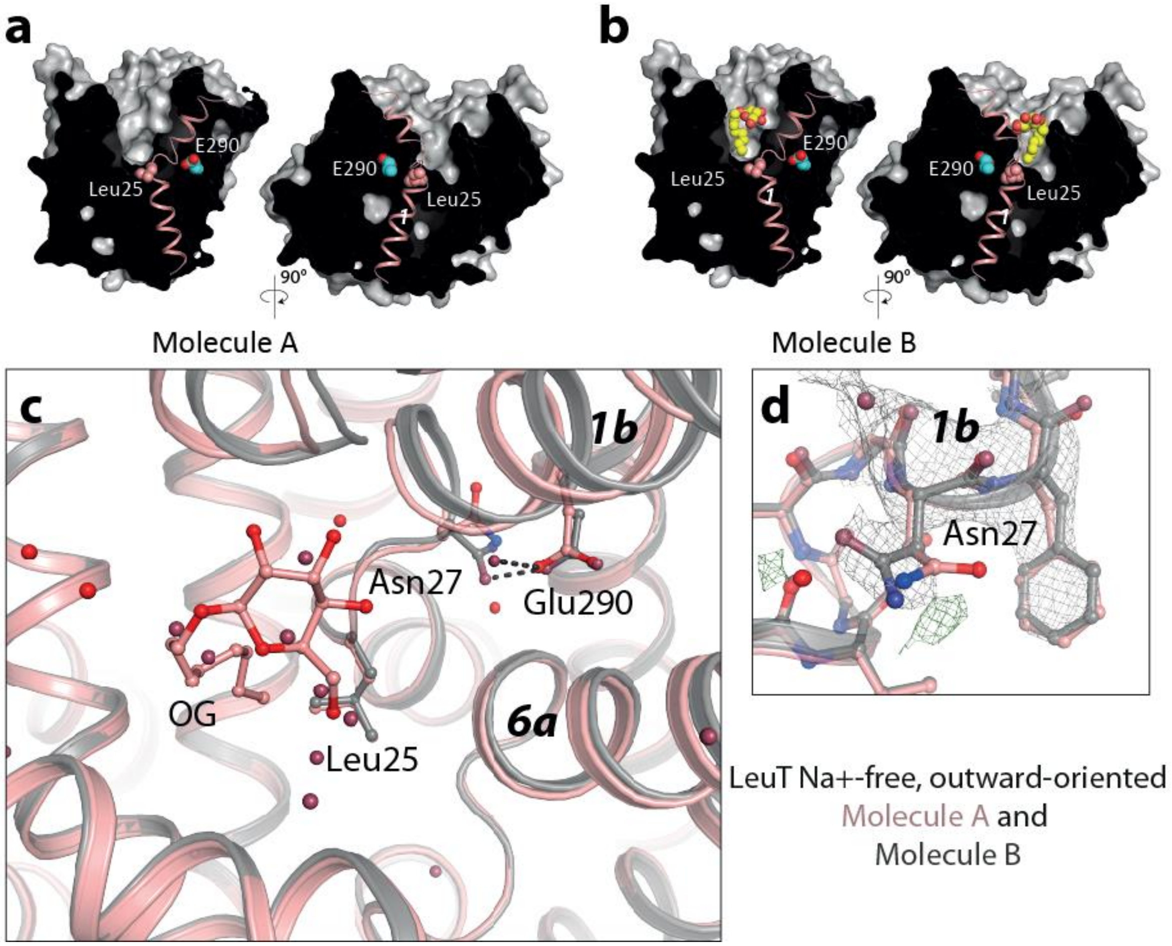

Supplementary Figure 2. Comparison of the extracellular cavity of the LeuT return step, Molecules A and B. a-b, Surface cut of LeuT return transport cycle crystal structure molecule A and B surface cuts with bound *n*-octyl-β-D-glucoside detergent molecule (yellow spheres) at the extracellular cavity of Molecule B (b). Leu25 (pink spheres) occludes Glu290 (cyan spheres) from the extracellular environment. **c**, Extracellular cavity overlay of Molecule A (grey, waters in magenta) and Molecule B (pink, waters in red). Asn27 (shown in sticks) interacts with Glu290 in Molecule A and is moved away in the more closed Molecule B. **d**, Molecule A Asn27 electron density map ($2F_o$-F_c map shown at 1 and F_o-F_c map at 3 r.m.s.d.) show clear density for the side chain, however extra F_o-F_c map features overlay with the superimposed Molecule B Asn27 rotamer and and an occluded water molecule, indicative of side chain flexibility.

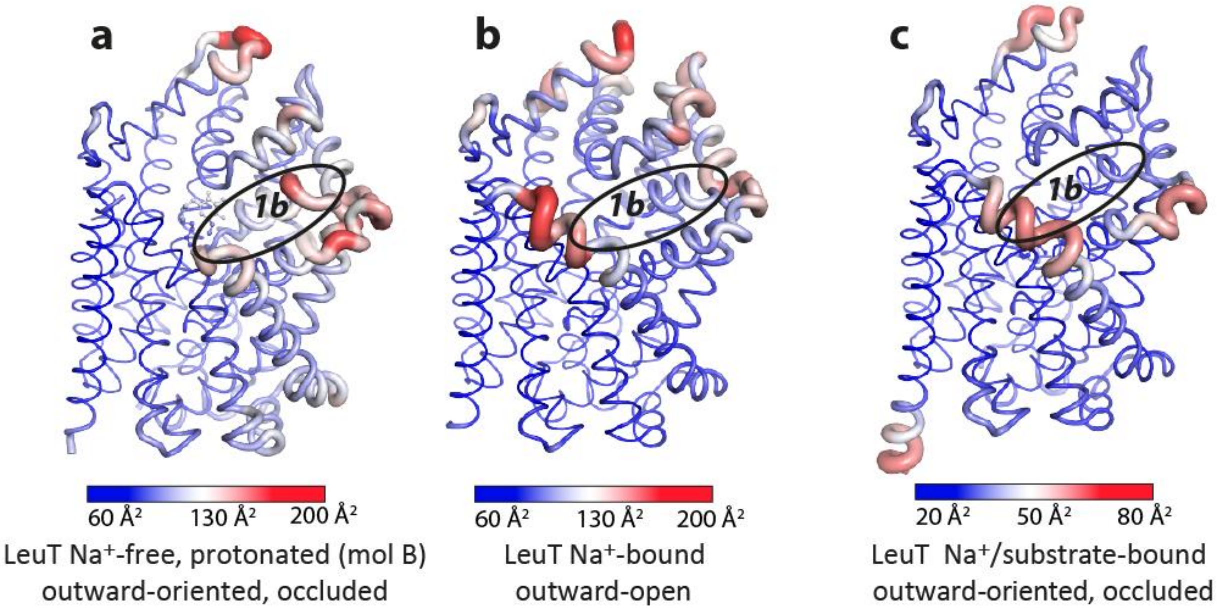

Supplementary Figure 3. Comparison of structural flexibility in LeuT outward-facing structures. Structural flexibility is indicated by the atomic displacement-coded cartoon putty thickness and color gradient from blue (low disorder) to red (high disorder): **a**, with average temperature factor values 94.3 Å^2 for the pH 6.5 structure Molecule B; **b**, 81.0 Å^2 for LeuT outward-open Na^+-bound state[1] (PDB entry code 3TT1); **c**, 27.7 Å^2 for LeuT outward-facing, Na+- and substrate-bound and occluded[2] (PDB entry code 2A65).

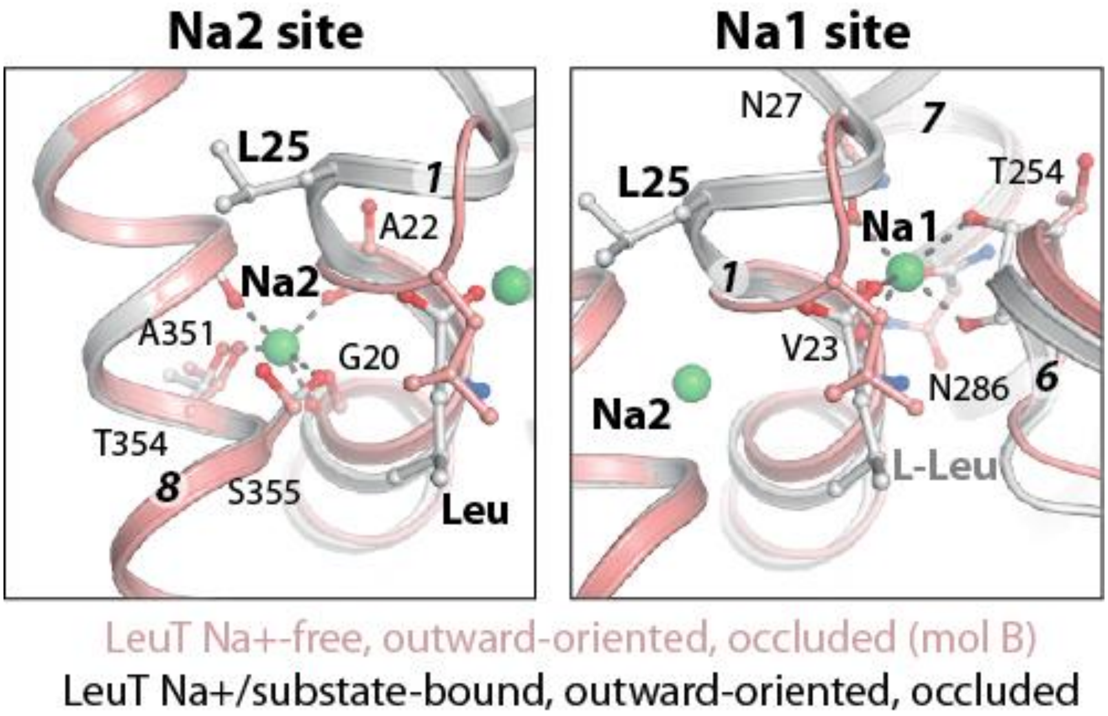

Supplementary Figure 4. Comparison of LeuT Na1 and Na2 sites in the outward-occluded, and the return states. Superposition of LeuT outward-facing return state (pink) with LeuT WT outward-facing occluded[2] (grey, PDB entry code 2A65) Na2 (left panel) and Na1 sites (right panel).

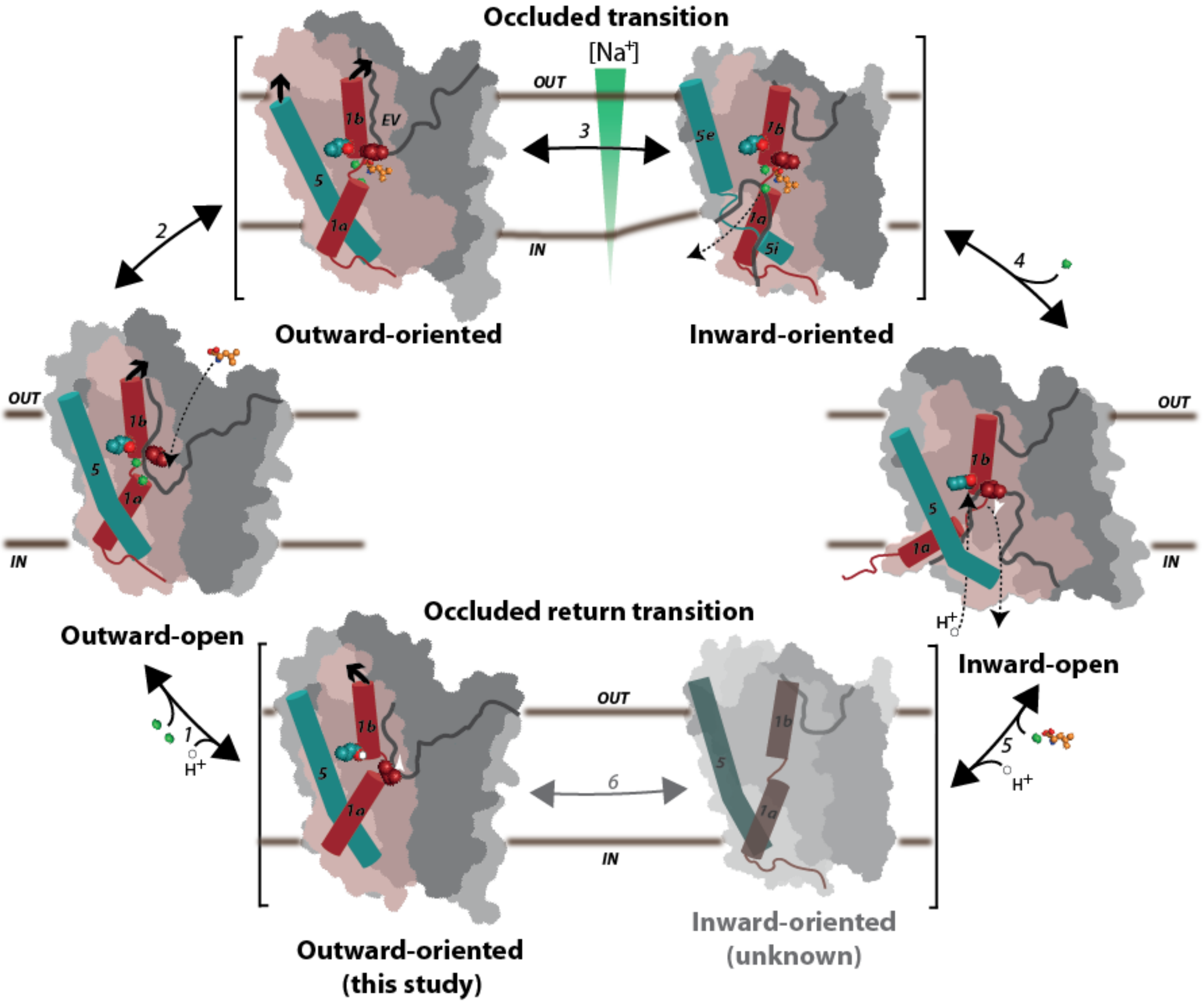

Supplementary Figure 5. In an outward-oriented return state Leu25 (red spheres) moves up (white arrow) to open the extracellular cavity for Na^+ ions to bind and H^+ release (*1*). Na^+ binding at the Na1 and Na2 sites stabilize the outward-open state and allows the substrate (orange spheres) to bind (dashed arrow), triggering substrate site occlusion (*2*). Further closure of the extracellular vestibule (EV) facilitates formation of an inward-oriented state where TM5 unwinding provides a solvation pathway for the Na2 site (*3*) and an opportunity for Na^+ to escape to the intracellular low-Na^+ environment (*4*). Na^+ release from Na2 allows TM5 to reform and TM1a to swing out to release the substrate with Na^+ from Na1 in the inward-open state. In the

inward-open state Glu290 (cyan spheres) becomes protonated (white sphere) and adopts a yet unknown inward-oriented occluded return state (*5*), which can switch to the outward-oriented H^+-occluded state described here (*6*). The scaffold domain is shown in dark grey, the bundle domain in pink, TM1 and TM5 as red and cyan cylinders, respectively, and Na^+ ions and amino acid substrate in green and orange spheres, respectively.

Supplementary Table 1. LeuT Glu290 pK_a value determined in different functional states: outward-open[1] (PDB entry code 3TT1), outward-oriented substrate-occluded[2] (PDB entry code 2A65), inward-open[1] (PDB entry code 3TT3), LeuT structures at pH 5.0 and 6.5 (this work). The calculations were done with PROPKA 3.1[3-6].

Structure	Predicted Glu290 pK_a	Crystallization conditions	Predicted Glu290 state
LeuT outward-facing occluded Leu- and Na^+-bound state	6.2	pH 7.0	deprotonated
LeuT outward-open state Na^+-bound state	5.4	pH 7.5	deprotonated
LeuT outward-open state, with removed Na^+ ions	6.9	pH 7.5	deprotonated
LeuT inward-open apo state	7.6	pH 7.6	partially protonated
LeuT return state at pH 5.0	7.9	pH 5.0	protonated
LeuT return state at pH 6.5 Molecule A	7.9	pH 6.5	protonated
LeuT return state at pH 6.5 Molecule B	7.8	pH 6.5	protonated

Supplementary Discussion

Combining all available structures of the NSS transporters we can describe a full transport cycle (Supplementary Fig. 6; Supplementary Movie 2): (*1*) From an initial outward-oriented state as presented here the transporter may release a counter-transported ion to the extracellular environment along with disengagement of $Leu25^{LeuT}$ from the substrate site. (*2*) Na^+-binding sites are then able to interact with the extracellular, high Na^+ concentration environment. (*3*) The transporter is now primed for high-affinity substrate binding that drives the transporter towards occlusion. (*4*) Closure of the hydrophobic, extracellular vestibule can now take place and is associated with unwinding and flexibility of TM5 and opening of a cytoplasmic solvation pathway leading to the Na2 site. (*5*) After Na^+ release from Na2, TM1a swings out and opens a large ion/substrate release pathway. (*6*) Upon substrate and Na^+ release the $Glu290^{LeuT}$ residue is protonated leading to closure of the inward-open state and presumably $Leu25^{LeuT}$ rotation into the S1 substrate-binding site that stabilizes a Na+ and substrate-free inward-oriented, occluded state (still unknown). Similar to the Na^+ and substrate-bound states this inward-oriented state may undergo a dynamic equilibrium exploring the outward-oriented, occluded state that allows Leu25 to rotate away from the substrate site to initiate another forward cycle.

Supplementary References

1. Krishnamurthy, H. & Gouaux, E. X-ray structures of LeuT in substrate-free outward-open and apo inward-open states. *Nature* **481**, 469-74 (2012).
2. Yamashita, A., Singh, S.K., Kawate, T., Jin, Y. & Gouaux, E. Crystal structure of a bacterial homologue of Na+/Cl--dependent neurotransmitter transporters. *Nature* **437**, 215-23 (2005).
3. Bas, D.C., Rogers, D.M. & Jensen, J.H. Very fast prediction and rationalization of pKa values for protein-ligand complexes. *Proteins* **73**, 765-83 (2008).
4. Li, H., Robertson, A.D. & Jensen, J.H. Very fast empirical prediction and rationalization of protein pKa values. *Proteins* **61**, 704-21 (2005).
5. Olsson, M.H.M., Søndergaard, C.R., Rostkowski, M. & Jensen, J.H. PROPKA3: Consistent Treatment of Internal and Surface Residues in Empirical pKa Predictions. *Journal of Chemical Theory and Computation* **7**, 525-537 (2011).
6. Søndergaard, C.R., Olsson, M.H.M., Rostkowski, M. & Jensen, J.H. Improved Treatment of Ligands and Coupling Effects in Empirical Calculation and Rationalization of pKa Values. *Journal of Chemical Theory and Computation* **7**, 2284-2295 (2011).
